# Prevalence and Associated Factors of Liver Enzyme Abnormalities Among Bangladeshi Women: A Cross-Sectional Study

**DOI:** 10.7759/cureus.57606

**Published:** 2024-04-04

**Authors:** Fatehatun Noor, Nusrat Jahan Shorovi, Sneha Sarwar, Tasmim Fahima Ahmad, Nisarga Bahar, Md. Musharraf Ashraf, Md. Ruhul Amin, Abu Ahmed Shamim, Johora Khatun Rima, M. Akhtaruzzaman

**Affiliations:** 1 Department of Food Science and Nutrition, Hajee Mohammad Danesh Science and Technology University, Dinajpur, BGD; 2 Institute of Nutrition and Food Science, University of Dhaka, Dhaka, BGD; 3 Center for Noncommunicable Diseases and Nutrition, BRAC James P Grant School of Public Health, BRAC University, Dhaka, BGD; 4 Department of Life Sciences, School of Environment and Life Sciences, Independent University, Bangladesh, Dhaka, BGD

**Keywords:** bangladesh, non-pregnant and non-lactating (npnl) women, non-alcoholic fatty liver disease (nafld), body mass index (bmi), aspartate aminotransferase (ast), alanine aminotransferase (alt)

## Abstract

Background

Liver enzyme abnormalities can indicate underlying liver health issues and are influenced by various factors. This study aimed to investigate the prevalence of liver enzyme abnormalities and their associated factors among nonpregnant and nonlactating (NPNL) women in Bangladesh.

Methodology

A cross-sectional study was conducted among 251 NPNL Bangladeshi women. Data on demographic, socioeconomic, and health-related variables were collected. Logistic regression analysis was used to determine the association between liver enzyme abnormalities and associated factors.

Results

The prevalence of liver enzyme abnormalities among participants was determined, with associated factors such as age, body mass index (BMI), monthly income, and food security status examined. Elevated alanine aminotransferase (ALT) and aspartate aminotransferase (AST) levels were observed in 54 (21.5%) and 47 (18.7%) of participants, respectively, with 116 (46.2%) exhibiting an AST/ALT ratio exceeding 1.00. Food insecurity was significantly associated with a higher prevalence of elevated ALT levels (24.4% vs. 8.7%, *P* = 0.02), as well as low monthly income (18.8%, 14.7% vs. 36.7%, *P* < 0.01) and higher BMI (11% vs. 27.7% and 25.6%, *P* = 0.02). Similar trends were observed for AST levels. Moreover, participants with a higher BMI exhibited significantly higher rates of at least one abnormal liver function enzyme (15.9% vs. 34.9%, *P* = 0.01). Logistic regression analysis revealed a significant association between abnormal liver enzyme levels and certain demographic and socioeconomic factors, specifically BMI and age.

Conclusions

This study provides insights into the prevalence of liver enzyme abnormalities and their associated factors among NPNL Bangladeshi women. The findings underscore the importance of addressing factors such as BMI and age in mitigating liver health issues in this population. Further research and targeted interventions are warranted to address these concerns effectively.

## Introduction

The liver plays a crucial role in detoxification, protein synthesis, and digestion [1]. It is also responsible for glucose synthesis and lipid metabolism [2]. With rapid urbanization and economic growth, the prevalence of overweight individuals has surged globally, including in Bangladesh [3]. Non-alcoholic fatty liver disease (NAFLD) is strongly linked to elevated body mass index (BMI) and is characterized by increased levels of liver function markers such as alanine aminotransferase (ALT) and aspartate aminotransferase (AST) [4,5]. Age and food insecurity are independent risk factors for NAFLD [6,7], and educational attainment and other socioeconomic factors may also influence its development [8].

The global prevalence of NAFLD was approximately 37% in 2019 [9]. However, the prevalence varies by country: India reported 38.6% [10], Japan 25.8% [11], China 29.8% [12], Korea 30.3% [13], and Sri Lanka 32.6% [14]. A study conducted in Bangladesh found an overall NAFLD prevalence of 33.86% across genders and urban-rural regions [6]. The prevalence of elevated ALT levels differs worldwide due to factors such as country, ethnicity, gender, and age. For instance, India reported a prevalence of 20% in healthy individuals [15], while in Bangladesh, the overall prevalence of elevated ALT and AST was found to be 18.8% and 21.6%, respectively. Female participants had a higher prevalence of elevated liver enzymes [16].

Despite these trends, there is a lack of research on the prevalence of elevated liver enzymes among nonpregnant and nonlactating (NPNL) women in Bangladesh and the factors associated with it. This study aims to address this gap by investigating the prevalence of elevated ALT and AST levels in NPNL Bangladeshi women and their association with BMI, food security, and age.

## Materials and methods

Study design and population

The study participants were selected from the *Nutrition, Health, and Demographic Survey of Bangladesh-2011* (NHDSBD-2011), which was a nationwide cross-sectional survey. NHDSBD-2011 provided a comprehensive understanding of the country's demographic profile, including population dynamics, health indicators, nutritional status, agricultural practices, and food security. The participants for the survey were chosen using a two-stage stratified random sampling technique based on the integrated multipurpose sample (IMPS) design, which was created using the Population and Housing Census 2001.

From the sampling units, households were carefully selected across the seven administrative divisions of Bangladesh. After a complete interview using a structured questionnaire, blood samples were collected from a subset of nonlactating and nonpregnant women aged between 17 and 49 years who were free from any diseases, for further investigation. Details of the study design have been described elsewhere [17].

For this study, a total of 251 women were selected from three districts (Dhaka, Khulna, and Chittagong), and their blood samples were analyzed to assess the prevalence of abnormal liver function enzymes.

Sociodemographic, anthropometric data, and blood collection

Data were collected using a structured, validated questionnaire [18], which included data on sociodemographic status, marital status, household food security, lifestyle, and nutrition. Nutritional status was measured using the anthropometric method. Blood samples were collected, and serum sample preparation was done by a medical team consisting of paramedics and physicians, following the guidelines outlined by Tuck et al. [19]. Trained personnel and interviewers with graduate-level education performed the anthropometric measurements using standardized protocols [20]. The weight of each participant, wearing minimum clothing, was taken to the nearest 100 g using a precision bathroom scale (Tanita HA-622, Tanita Corporation, Tokyo, Japan), while height, measured barefoot, was recorded to the nearest 0.1 cm using a height scale.

Biochemical analysis

A total of 251 samples were analyzed to determine the levels of ALT and AST in women. Participants in the study were required to fast for a minimum of eight hours before providing a blood sample. Approximately 6 mL of venous blood was collected using disposable syringes and transferred to plain dry vacutainer tubes. The serum samples were then prepared and stored in a freezer at -20 °C. To maintain sample integrity during transportation, the serum samples were transported to Dhaka from the collection areas using dry ice in an insulated box, ensuring temperature control and sample preservation.

The ALT and AST enzyme activity levels were analyzed using the DIALAB GmbH semi-auto biochemistry analyzer DTN-405 (Austria). To ensure accuracy, every 20th sample was re-analyzed, and 12 samples were analyzed twice to estimate the coefficient of variation (CV).

The CV was calculated using the formula CV = (Standard Deviation [SD]/Mean estimated value of the sample) * 100. The average CV for ALT and AST from the repeated sample analyses was 12.82% and 6.37%, respectively.

Definition of outcome variables

Impaired liver function was defined as ALT > 34 U/L and AST > 31 U/L in women, as established by prior research [21,22]. An AST-to-ALT ratio >1 was also considered indicative of impaired liver function [23].

Exposure variables

Demographic and socioeconomic data were presented categorically. Due to limited representation of adolescents and elderly women, respondent age was divided into three groups: 23 years and under, 24 to 28 years, and 29 years and older. Marital status was classified as unmarried or married. Household information included monthly family income, expenditure on food, and access to electricity. Monthly family income was stratified into three groups: 8,000 Bangladeshi Taka (BDT) and below, 8,001-15,000 BDT, and 15,001 BDT and above. Monthly expenditure on food was divided into three categories: low expenditure (4,500 BDT), medium expenditure (4,501-6,000 BDT), and high expenditure (6,001+ BDT). Household food security was assessed by asking respondents about experiences of food shortage throughout the year, resulting in two groups: those who had never encountered food insecurity and those who had experienced occasional or frequent food insecurity.

BMI was categorized into three groups using terciles (<=19.25, 19.26-21.98, and >=21.99). Additionally, BMI was classified using Asian cutoff points: underweight (<18.5 kg/m^2^), normal weight (18.5-23 kg/m^2^), overweight (23-27.4 kg/m^2^), and obese (≥27.5 kg/m^2^) [24], as well as the World Health Organization (WHO) cutoff points: underweight (<18.5 kg/m^2^), normal weight (18.5-24.9 kg/m^2^), overweight (25-29.9 kg/m^2^), and obese (≥30 kg/m^2^) [25].

Ethical approval

The study received approval from the Ethical Board of the Faculty of Biological Science, University of Dhaka (approval no. 10/Bio. Sci./2015-2016, on October 25, 2015). Written consent was obtained from all participants before data and sample collection.

Statistical analysis

All statistical analyses were performed using the statistical software IBM SPSS Statistics for Windows, Version 26.0 (IBM Corp., Armonk, NY). Descriptive analyses were conducted for all variables, with median values and interquartile ranges (IQRs) reported due to the non-normal distribution of the samples. The chi-square test was used to assess associations between various factors (including food security, BMI, age, and monthly family income) and indicators of liver health (AST level, ALT level, and AST/ALT ratio). Variables showing significance levels <0.2 in their association were included in the logistic regression model.

## Results

Table 1 presents an overview of basic characteristics among the study population (*n* = 251), delineating frequencies and percentages for each variable. Among the participants, 90 (35.9%) of NPNL women were aged between 24 and 28 years, with a median age of 26 years. A total of 237 (94.4%) participants were married. Access to electricity was reported by 111 (44.4%) of the respondents. Regarding monthly family income, the largest proportion of participants (96, 38.3%) fell within the range of 8,001 to 15,000 BDT, with a median income of 10,000 BDT. In terms of monthly food expenditure, 91 (36.3%) participants spent between 4,501 and 6,000 BDT, with a median food expenditure of 5,000 BDT. The majority of the respondents (205, 81.7%) reported never experiencing food insecurity, while 18 (18.8%) acknowledged occasional or frequent challenges. The mean levels for AST and ALT found among the participants were 26.12 and 26.06 U/L respectively. The median levels for AST and ALT were 24.21 and 26.13 U/L, respectively.

**Table 1:** Basic characteristics of the study participants (n = 251). BDT, Bangladeshi Taka; BMI, body mass index; ALT, alanine aminotransferase; AST, aspartate aminotransferase; IQR, interquartile range

Variables		*n* (%)
Age (years)	≤23	79 (31.5)
	24-28	90 (35.9)
	≥29	82 (32.7)
	Median (IQR)	26 (22-30)
Marital status	Unmarried	14 (5.6)
	Married	237 (94.4)
Monthly expenditure on food (BDT)	≤4,500	84 (33.5)
	4,501-6,000	91 (36.3)
	≥6,001	76 (30.3)
	Median (IQR)	5,000 (4,000-7,000)
Monthly family income (BDT)	≤8,000	96 (38.2)
	8,001-15,000	95 (37.8)
	≥15,001	60 (23.9)
	Median (IQR)	10,000 (7,000-15,000)
Access to electricity	Yes	114 (45.4)
	No	137 (54.6)
Food insecurity	Never experienced	205 (81.7)
	Sometime/often	46 (18.3)
BMI	≤19.25	82 (32.7)
	19.26-21.98	83 (33.1)
	≥21.99	86 (34.3)
	Median (IQR)	20.65 (18.72-23.24)
Serum ALT	Mean ± SD	26.12 ± 12.67
	Median (IQR)	26.13 (17.00-33.00)
Serum AST	Mean ± SD	26.06 ± 14.72
	Median (IQR)	24.21 (18.00-30.72)

**Figure 1:**
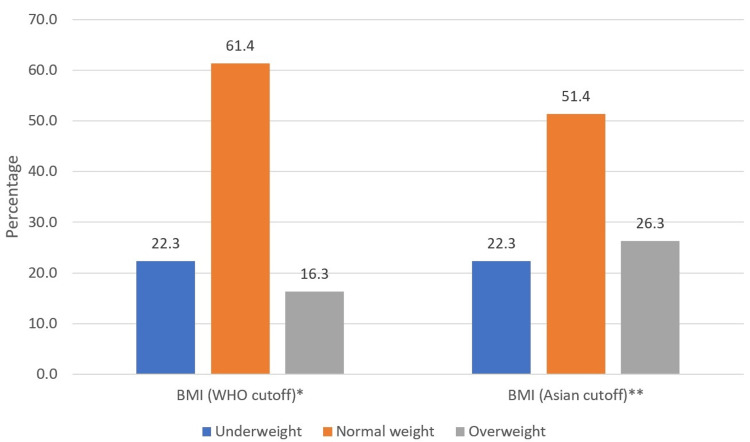
Nutritional status of the participants according to the World Health Organization (WHO) and Asian cutoffs. ^*^WHO cutoff for body mass index (BMI): underweight (<18.5 kg/m^2^), normal weight (18.5-24.9 kg/m^2^), overweight (≥25 kg/m^2^). ^**^Asian cutoff: underweight (<18·5 kg/m^2^), normal weight (18·5-23 kg/m^2^), and overweight (≥23 kg/m^2^).

Figure 1 compares the distribution of BMI categories among participants using both the WHO and Asian cutoff points. At the WHO cutoff, the majority were of normal weight (154, 61.4%), followed by overweight (41, 16.3%) and underweight (56, 22.3%). However, applying the Asian cutoff resulted in a lower percentage of normal weight (129, 51.4%), with more overweight (66, 26.3%) and underweight (56, 22.3%) participants.

**Figure 2:**
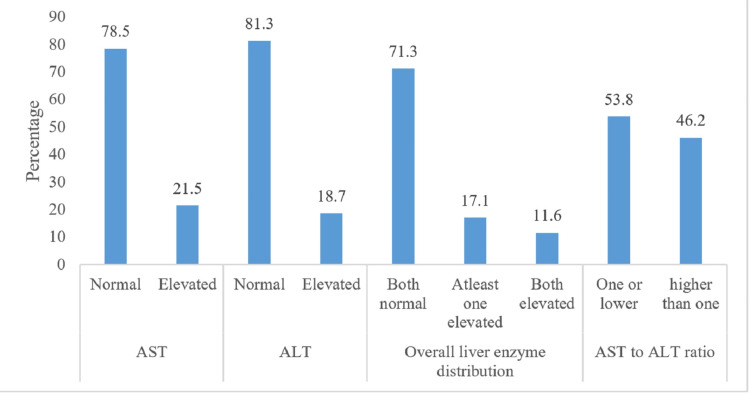
Status of the participants by liver enzyme levels. Elevated ALT and AST levels were defined as serum ALT > 34 U/L and serum AST > 31 U/L in women, respectively. ALT, alanine aminotransferase; AST, aspartate aminotransferase

Figure 2 illustrates that the majority of participants had normal levels of ALT and AST, with 197 (78.5%) and 204 (81.3%) falling within normal ranges, respectively. Elevated levels were observed in 54 (21.5%) participants for ALT and 47 (18.7%) for AST. Additionally, 116 (46.2%) participants exhibited an AST/ALT ratio exceeding 1.00. Notably, 179 (71.3%) participants had normal levels for both liver markers, while 43 (17.1%) had at least one marker elevated, and 29 (11.6%) had elevated levels for both markers.

Table 2 depicts the relationship between abnormal liver function markers and various demographic and socioeconomic factors. A significant association was observed between food insecurity and a higher prevalence of elevated ALT levels compared to food-secure counterparts (24.4% vs. 8.7%, *P* = 0.02). Similarly, a significant association was observed between elevated ALT and low monthly income (18.8%, 14.7% vs. 36.7%, *P *< 0.01) as well as BMI (11% vs. 27.7% and 25.6%, *P* = 0.02). Similar trends were noted for AST levels. Additionally, participants with a higher BMI exhibited a significantly higher rate of at least one abnormal liver function enzyme (15.9% vs. 34.9%, *P* = 0.01).

**Table 2:** Association between sociodemographic factors and liver enzyme status among the participants. ^*^P-value < 0.05. BDT, Bangladeshi Taka; BMI, body mass index; AST, aspartate aminotransferase; ALT, alanine aminotransferase

Categories		Food security status		Chi-square *P* -value	Age (years)			Chi-square *P* -value	Monthly family income (BDT)			Chi-square *P* -value	BMI			Chi-square *P* -value
		Food secure, *n* (%)	Food insecure (usually/sometimes), *n* (%)		<=23, *n* (%)	24 – 28, *n* (%)	29+, *n* (%)		<=8,000, *n* (%)	8,001-15,000, *n* (%)	15,001+, *n* (%)		<=19.27, *n* (%)	19.26-21.10, *n* (%)	21.99+, *n* (%)	
ALT	Normal	155 (75.6)	42 (91.3)	0.02*	66 (83.5)	64 (71.1)	67 (81.7)	0.10	78 (81.3)	81 (85.3)	38 (63.3)	<0.01*	73 (89.0)	60 (72.3)	64 (74.4)	0.02*
	Elevated	50 (24.4)	4 (8.7)		13 (16.5)	26 (28.9)	15 (18.3)		18 (18.8)	14 (14.7)	22 (36.7)		9 (11.0)	23 (27.7)	22 (25.6)	
AST	Normal	165 (80.5)	39 (84.8)	0.50	70 (88.6)	65 (72.2)	69 (84.1)	0.02*	81 (84.4)	78 (82.1)	45 (75.0)	0.33	71 (86.6)	67 (80.7)	66 (76.7)	0.26
	Elevated	40 (19.5)	7 (15.2)		9 (11.4)	25 (27.8)	13 (15.9)		15 (15.6)	17 (17.9)	15 (25.0)		11 (13.4)	16 (19.3)	20 (23.3)	
Abnormal liver function markers	Both normal	142 (68.5)	37 (80.0)	0.13	63 (79.7)	58 (64.4)	58 (70.7)	0.09	74 (77.1)	70 (73.7)	35 (58.3)	0.03	69 (84.1)	54 (65.1)	56 (65.1)	0.01*
	At least one elevated	63 (31.5)	9 (20.0)		16 (20.3)	32 (35.6)	24 (29.3)		22 (22.9)	25 (26.3)	25 (41.7)		13 (15.9)	29 (34.9)	30 (34.9)	
AST/ALT	1 or low	117 (57.1)	18 (39.1)	0.03*	40 (50.6)	52 (57.8)	43 (52.4)	0.62	55 (57.3)	43 (45.3)	37 (61.7)	0.09	40 (48.8)	44 (53.0)	51 (59.3)	0.39
	Higher than 1	88 (42.9)	28 (60.9)		39 (49.4)	38 (42.2)	39 (47.6)		41 (42.7)	52 (54.7)	23 (38.3)		42 (51.2)	39 (47.0)	35 (40.7)	

The logistic regression analysis highlighted significant associations between abnormal liver markers and BMI, adjusted for age, monthly family income, and food insecurity (Table 3). Notably, individuals with a BMI between 19.26 and 21.98 had a significantly higher likelihood of abnormal liver enzyme markers compared to those with a BMI <= 19.25 (adjusted odds ratio [AOR] 2.81, *P* = 0.01). This association persisted even after adjusting for other variables. Furthermore, individuals aged 24-28 years showed significantly higher odds of abnormal liver markers compared to those aged 23 or younger (almost 2.09 times higher, *P* = 0.04). These findings underscore the relevance of BMI and age as independent predictors of abnormal liver markers (Table 3).

**Table 3:** Logistic regression analysis of associations between abnormal liver enzyme levels and anthropometric and socioeconomic factors. ^*^P-value < 0.05. BDT, Bangladeshi Taka; OR, odds ratio

Abnormal liver markers		Crude OR	*P*-value	Adjusted OR	*P-*value
BMI	<=19.25	Reference	0.01	Reference	0.03
	19.26-21.98	2.85 (1.35, 6.00)	0.01	2.81 (1.28, 6.15)	0.01*
	21.99+	2.84 (1.36, 5.96)	0.01	2.23 (0.98, 5.07)	0.06
Age (years)	<=23	Reference	0.09	Reference	0.09
	24-28	2.17 (1.08, 4.37)	0.02	2.09 (1.01, 4.35)	0.04*
	29+	1.62 (0.79, 3.37)	0.18	1.18 (0.54, 2.57)	0.67
Food insecurity	Never	Reference		Reference	
	Sometimes/often	0.54 (0.25, 0.20)	0.13	0.69 (0.29, 1.60)	0.38
Monthly family income (BDT)	<=8,000	Reference	0.04	Reference	0.17
	8,001-15,000	1.201 (0.62, 2.32)	0.59	0.95 (0.47, 1.94)	0.89
	15,001+	2.403 (1.19, 4.84)	0.014	1.88 (0.84, 4.21)	0.12

## Discussion

The primary aim of this study was to assess the prevalence of impaired liver function enzymes, including AST, ALT, and the AST-to-ALT ratio, among 251 NPNL Bangladeshi women. Our findings revealed that approximately one-third of the participants exhibited elevated levels of at least one liver enzyme. Notably, our analysis indicated a significant association between elevated liver enzymes and BMI, even after adjusting for age, monthly income, and food security status.

In our study, we observed the mean values of ALT and AST as 26.12 and 26.06 U/L, respectively. These findings were parallel to the studies of other subcontinental countries. For example, in India, the mean values of ALT and AST among healthy women were observed to be 22.0 and 24.0 U/L, respectively [26]. Similarly, in Pakistan, the mean ALT value was reported as 30.12 U/L [27]. The slight difference observed can be attributed to population variations.

Food security is a crucial factor for good health and nutritional well-being. In our study, we reported an association between the food security status and the abnormal liver function markers. Mainly, food insecurity is prevalent among low-income families, which increases their vulnerability to NAFLD. It is seen that food-insecure women from these communities frequently turn to energy-dense, high-fat foods that lack essential nutrients. Furthermore, this population is also subjected to financial insecurity and a lack of access to proper healthcare, both of which can lead to poor health and abnormal liver function markers [28].

Macek et al. highlighted the intricate relationship between BMI and age, noting that as age increases, BMI tends to rise as well [29]. It's noteworthy that our study stands out with a considerably lower median age (25 years) among participants compared to earlier investigations, which probably resulted in a lower median BMI (20.6 kg/m²) observed in our study. We found that individuals with a higher age and BMI exhibited a twofold increased likelihood of abnormal liver markers compared to those with a lower BMI. This trend remained significant even after adjusting for other variables. These results align with previous research conducted among Bangladeshi women [22] and the Australian adult population [30].

Most of the liver enzyme abnormalities are often linked with high BMI [22,31]. Excessive weight or high BMI often leads to NAFLD. NAFLD involves the accumulation of excess fat in liver cells, a condition known as hepatic steatosis. Non-alcoholic steatohepatitis (NASH), a progressive form of NAFLD, is characterized by oxidative stress, inflammation, and liver cell damage, leading to elevated AST and ALT levels in the blood [31]. Consequently, such elevated liver enzymes may serve as indicators of NAFLD presence among the participants of this study. If NAFLD is not managed timely, it may trigger fibrosis, ultimately resulting in liver cirrhosis [31].

Strengths and limitations

This study had several strengths. The study explored the overall prevalence and association of liver enzymes (ALT and AST) with the nutritional status and socioeconomic conditions of the participants. Conducted using a cross-sectional design, the study provides valuable insights although its ability to establish causal relationships is constrained. Given that a significant portion of participants fell within the normal weight category, the study adopted both established Asian or WHO BMI cutoffs and terciles to ensure a comprehensive analysis. Similarly, age was categorized into terciles rather than relying solely on established cutoffs, enabling thorough exploration of its association with abnormal liver function markers. However, the study had some limitations. Important confounding variables such as dietary habits and physical activity were not accounted for in the analysis. Moreover, the frozen storage (-20 °C) and subsequent thawing of serum samples at the Institute of Nutrition and Food Science laboratory may have impacted the levels of liver enzymes. Nevertheless, research by Leu et al. revealed high relative agreement in the validity of biomarkers in frozen serum samples stored for extended periods, supporting their utility in longitudinal epidemiological studies [32]. Further research should include a wider range of factors and diverse population groups to enhance our understanding of the determinants of liver health and to guide targeted interventions.

## Conclusions

In summary, the study found that one-third of participants had abnormal hepatic enzyme levels. It was observed that increased BMI was a strong predictor of this, even after taking related variables into account. Therefore, interventions should focus on raising awareness among women about the connection between elevated BMI and liver health. These findings have important implications for evidence-based policies and interventions aimed at improving well-being and preventing diseases in NPNL women.

## References

[1] Iluz-Freundlich D, Zhang M, Uhanova J, Minuk GY (2020). The relative expression of hepatocellular and cholestatic liver enzymes in adult patients with liver disease. Ann Hepatol.

[2] Maruyama S, Hirayama C, Yamamoto S (2001). Red blood cell status in alcoholic and non-alcoholic liver disease. J Lab Clin Med.

[3] Tanwi TS, Chakrabarty S, Hasanuzzaman S, Saltmarsh S, Winn S (2019). Socioeconomic correlates of overweight and obesity among ever-married urban women in Bangladesh. BMC Public Health.

[4] Patell R, Dosi R, Joshi H, Sheth S, Shah P, Jasdanwala S (2014). Non-alcoholic fatty liver disease (NAFLD) in obesity. J Clin Diagn Res.

[5] Andy SY, Keeffe EB (2003). Elevated AST or ALT to nonalcoholic fatty liver disease: accurate predictor of disease prevalence?. Am J Gastroenterol.

[6] Alam S, Hossain M, Azam G (2017). Nonalcoholic fatty liver disease: a new frontier for hepatology in Bangladesh and a call for action to combat. J Bangladesh Coll Phys Surg.

[7] Golovaty I, Tien PC, Price JC, Sheira L, Seligman H, Weiser SD (2020). Food insecurity may be an independent risk factor associated with nonalcoholic fatty liver disease among low-income adults in the United States. J Nutr.

[8] Koutny F, Aigner E, Datz C (2023). Relationships between education and non-alcoholic fatty liver disease. Eur J Intern Med.

[9] Le MH, Yeo YH, Li X (2022). 2019 Global NAFLD prevalence: a systematic review and meta-analysis. Clin Gastroenterol Hepatol.

[10] Shalimar Shalimar, Elhence A, Bansal B, Gupta H, Anand A, Singh TP, Goel A (2022). Prevalence of non-alcoholic fatty liver disease in India: a systematic review and meta-analysis. J Clin Exp Hepatol.

[11] Fujii H, Suzuki Y, Sawada K (2023). Prevalence and associated metabolic factors of nonalcoholic fatty liver disease in the general population from 2014 to 2018 in Japan: a large-scale multicenter retrospective study. Hepatol Res.

[12] Wu Y, Zheng Q, Zou B (2020). The epidemiology of NAFLD in Mainland China with analysis by adjusted gross regional domestic product: a meta-analysis. Hepatol Int.

[13] Im HJ, Ahn YC, Wang JH, Lee MM, Son CG (2021). Systematic review on the prevalence of nonalcoholic fatty liver disease in South Korea. Clin Res Hepatol Gastroenterol.

[14] Dassanayake AS, Kasturiratne A, Rajindrajith S (2009). Prevalence and risk factors for non-alcoholic fatty liver disease among adults in an urban Sri Lankan population. J Gastroenterol Hepatol.

[15] Amarapurkar D, Kamani P, Patel N (2007). Prevalence of non-alcoholic fatty liver disease: population based study. Ann Hepatol.

[16] Kathak RR, Sumon AH, Molla NH (2022). The association between elevated lipid profile and liver enzymes: a study on Bangladeshi adults. Sci Rep.

[17] Akhtaruzzaman M, Khan NI, Islam SN (2013). Nutrition, Health and Demographic Survey of Bangladesh-2011. https://pdf.usaid.gov/pdf_docs/PBAAC556.pdf.

[18] National Institute of Population Research and Training (NIPORT), Mitra and Associates, and ICF International (2013). Bangladesh Demographic and Health Survey 2011. http://dhsprogram.com/pubs/pdf/FR265/FR265.pdf..

[19] Tuck MK, Chan DW, Chia D (2009). Standard operating procedures for serum and plasma collection: early detection research network consensus statement standard operating procedure integration working group. J Proteome Res.

[20] Gibson RS (2005). Principles of Nutritional Assessment. https://books.google.com.bd/books?id=lBlu7UKI3aQC&lpg=PR11&ots=RYLCQQ4vrB&dq=%20Gibson%20RS.%20Principles%20of%20nutritional%20assessment.%20Oxford%20university%20press%2C%20USA%3B%202005.&lr&pg=PR15#v=onepage&q=Gibson%20RS.%20Principles%20of%20nutritional%20assessment.%20Oxford%20university%20press,%20USA;%202005.&f=false.

[21] Schumann G, Klauke R (2003). New IFCC reference procedures for the determination of catalytic activity concentrations of five enzymes in serum: preliminary upper reference limits obtained in hospitalized subjects. Clin Chim Acta.

[22] Ali N, Sumon AH, Fariha KA (2021). Assessment of the relationship of serum liver enzymes activity with general and abdominal obesity in an urban Bangladeshi population. Sci Rep.

[23] Nyblom H, Björnsson E, Simrén M, Aldenborg F, Almer S, Olsson R (2006). The AST/ALT ratio as an indicator of cirrhosis in patients with PBC. Liver Int.

[24] Tan KCB (2004). Appropriate body-mass index for Asian populations and its implications for policy and intervention strategies. Lancet.

[25] (1998). Clinical guidelines on the identification, evaluation, and treatment of overweight and obesity in adults-the evidence report. National Institutes of Health. Obes Res.

[26] Yadav D, Mishra S, Gupta M, John PJ, Sharma P (2013). Establishment of reference interval for liver specific biochemical parameters in apparently healthy north Indian population. Indian J Clin Biochem.

[27] Saeed M, Waheed U, Wazeer A, Saba N (2023). Do we need Pakistan-specific reference ranges in laboratory medicine?. J Lab Physicians.

[28] Yadlapati S, Christian VJ, Shah A (2023). Fatty liver disease and food insecurity: excess in scarcity. Curr Nutr Rep.

[29] Macek P, Terek-Derszniak M, Biskup M, Krol H, Smok-Kalwat J, Gozdz S, Zak M (2020). Assessment of age-induced changes in body fat percentage and BMI aided by Bayesian modelling: a cross-sectional cohort study in middle-aged and older adults. Clin Interv Aging.

[30] Adams LA, Knuiman MW, Divitini ML, Olynyk JK (2008). Body mass index is a stronger predictor of alanine aminotransaminase levels than alcohol consumption. J Gastroenterol Hepatol.

[31] Shil BC, Saha M, Ahmed F, Dhar SC (2015). Nonalcoholic fatty liver disease: study of demographic and predictive factors. Euroasian J Hepatogastroenterol.

[32] Leu M, Mehlig K, Hunsberger M (2015 ). Quality assessment of 25 (OH) D, insulin, total cholesterol, triglycerides, and potassium in 40-year-old frozen serum. Epidemiol Res Int.

